# Prognostic value of serum soluble ST2 in stable coronary artery disease: a prospective observational study

**DOI:** 10.1038/s41598-021-94714-3

**Published:** 2021-07-26

**Authors:** Hack-Lyoung Kim, Jung Pyo Lee, Nathan Wong, Woo-Hyun Lim, Jae-Bin Seo, Joo-Hee Zo, Myung-A Kim, Sang-Hyun Kim

**Affiliations:** 1 Division of Cardiology, Department of Internal Medicine, Seoul National University College of Medicine, Boramae Medical Center, 20, Boramae-ro 5-gil, Dongjak-gu, Seoul, 07061 Korea; 2 Division of Nephrology, Department of Internal Medicine, Seoul National University College of Medicine, Boramae Medical Center, Seoul, Korea; 3 Heart Disease Prevention Program, Division of Cardiology, University of California, Irvine, CA USA

**Keywords:** Biomarkers, Cardiology

## Abstract

The role of ST2 in stable coronary artery disease (CAD) has not yet been well defined. This study was performed to investigate baseline serum soluble ST2 (sST2) level can predict clinical outcomes in patients with stable CAD. A total of 388 consecutive patients with suspected CAD (65 years and 63.7% male) in stable condition referred for elective invasive coronary angiography (ICA) was prospectively recruited. Major adverse cardiovascular event (MACE), including cardiac death, non-fatal myocardial infarction, coronary revascularization (90 days after ICA), and ischemic stroke during clinical follow-up was assessed. Most of the patients (88.0%) had significant CAD (stenosis ≥ 50%). During median follow-up of 834 days, there was 29 case of MACE (7.5%). The serum sST2 level was significantly higher in patients with MACE than those without (47.3 versus 30.6 ng/ml, *P* < 0.001). In multiple Cox regression model, higher sST2 level (≥ 26.8 ng/ml) was an independent predictor of MACE even after controlling potential confounders (hazard ratio, 13.7; 95% confidence interval 1.80–104.60; *P* = 0.011). The elevated level of baseline sST2 is associated with an increased risk of adverse clinical events in stable CAD patients. Studies with larger sample size are needed to confirm our findings.

## Introduction

Coronary artery disease (CAD) is the leading cause of mortality and morbidity globally^1^. Despite improvements in diagnosis and therapy, the prognosis of CAD is still poor, and CAD has become a significant burden on our society^2^. In order to reduce CAD associated risk, individualized therapy based on the risk stratification is clinically important. Although several biomarkers such as cardiac troponin, natriuretic peptide and C-reactive protein have been developed for the risk stratification of CAD^3^, prognostic information gained from these tests only partially explains the increased risk for CAD, and there continue to be unmet needs for additional biomarkers with more precise estimate of risk. ST2, an interleukin-1 receptor family member, is induced by the mechanical stretch of myocardium, and its expression is markedly increased in infarcted myocardium^4^. Following series of studies have shown that increased serum level of soluble ST2 (sST2) predict worse prognosis in patients with heart failure^5–7^ and acute myocardial infarction (AMI)^8–10^. However, there are limited data on whether the association between sST2 and clinical outcomes also can be extended to those with stable CAD. Therefore, this study was performed to investigate the prognostic value of ST2 in patients with stable CAD. Based on the strong prognostic value of sST2 in heart failure and AMI, we hypothesized that measurement of sST2 would be helpful even in stable CAD patients by identifying high risk patients who benefit from more intensified treatment strategies.

## Methods

### Study patients

This single-center study was performed at Boramae Medical Center (Seoul, Korea). Between January 2013 and December 2015, consecutive patients who underwent elective invasive coronary angiography (ICA) were prospectively recruited. Coronary angiography was performed for suspected CAD. A total of 466 paitents were initially screened, however, 78 patients with following conditions were exclued: (1) AMI, (2) ongoing chest pain, (3) unstable vital signs, (4) left ventricular ejection fraction < 50% or clinical heart failure syndrom, (5) the presence of regional wall motion abnormalies, (6) valvular dysfunction greater mild degree, and (7) the presence of pericardial effusion. Finally, a total of 388 patients were analyzed in this study. This study conforms to the ethical guidelines of the Declaration of Helsinki, and the study protocol was approved by the Institutional Review Board (IRB) of Boramae Medical Center (Seoul, Korea) (IRB number: 16-2015-161). All subjects provided written informed consent prior to their participation in the study.

### Data collection

The height and weight were measured on the day of hospital admission by a nurse, and BMI was calculated using wieght and height (kg/m^2^). Hypertension, diabetes mellitus and dyslipidemia were diagnosed based on a previous history of diagnosis made by doctors or current medications controlling them. Current smokers were defined as those who regularly smoked in the last year. After overnight fasting for 12 h, blood was collected from antecubital vein, and the blood levels following parameters were masured: total cholesterol, low-density lipoprotein (LDL) and high-density lipoprotein (HDL) cholesterol, and triglyceride and creatinine. The estimated glomerular filtration rate (eGFR) was calculated using the following the Modification of Diet in Renal Disease (MDRD) study formula: 175 × serum creatinine^−1.154^ × age^−0.203^ (× 0.742, if woman). Left ventricular ejection fraction was measured by Simpson’s biplane method using tranthoracic echocardiography. Concomitant cardiovascular medications taken at study entry including atniplatelet, beta-blocker, renin-angiotensin system blocker, nitrate and statin were also reviewed.

### ICA

Coronary angiography and percutaneous coronary intervention were performed in accordiance with current guidelines^11^. After ICA with or without revascularization, all patients received the standard medical treatment at the discretion of the attending physician. Luminal narowwing more than 50% of the major epicardial coronary artery or main branches with diameter ≥ 2 mm was considered significant coronary artery stenosis. CAD extent was classified as 1-, 2- or 3-vessel diseasebased on the number of coronary arteris with significant stenosis. Left main stenosis more than 50% was considered as 2-vessel disease. Cardiac catheterization was performed by a single experienced interventional cardiologist.

### Blood tests

Before the day of ICA, patients were overnight fast for at least 12 h. After insertion of arterial sheath to radial or femoral arteries, 20 mL of arterial blood was drawn from the sheath in the supine position just before ICA. The drawn arterial blood was immediately centrifuged at 3000 rpm for 15 min, and the separated serum was stored at − 70 °C until used for analysis. Using a commercially available kit (The Presage® ST2 Assay, Critical Diagnostics, San Diego, CA, USA), the serum levels of sST2 were measured. The minimum detectable concentration of sST2 was 3.1–200.0 ng/ml. The intra-assay and inter-assay coefficients of variations for sST2 were 5.1% and 5.2%, respectively.

### Clinical events

After index hospitalization, patients were followed-up every 3 month. Composite events, termed major adverse cardiac events (MACE), including cardiac death, non-fatal AMI, coronary revascularization (PCI [percutaneous coronary intervention] or coronary bypass surgery), and ischemic stroke were assessed. All outcome data was evaluated by an expert physician who was blinded to sST2 concentration. Cardiac death was defined as a death from ventricular arrhythmia, acute coronary syndrome, heart failure and sudden death. AMI was defined based on patient’s symptom, electrocardiographic changes and elevated cardiac enzyme. In order to exclude, test-driven procedure, PCI or coronary bypass surgery 90 days after ICA was considered as MACE. Ischemic stroke was defined as an episode of neurological dysfunction caused by focal cerebral infarction, which was demonstrated by brain imaging study.

### Statistical analysis

Continuous variables are presented as mean ± standard deviation (SD), and categorical variables are expressed as counts and percentages. Continuous variables were compared using Student’s t-test, and categorical variables are compared using The Fischer exact test or the *χ*^2^ test between groups with and without MACE. Receiver operating characteristic (ROC) curve analysis was used to get a cut-off value of sST2 predicting MACE. MACE free survival curves according to sST2 levels were generated using Kaplan–Meier survival estimates, and the differences in survival rates were compared using the log-rank test. Multivariable Cox proportional hazard model was used to analyze the effect of sST2 and several potential confounders on MACE, and hazard ratio and 95% confidence interval were reported. Confounders adjusted in multivariable model include age, sex, BMI, hypertension, diabetes mellitus, dyslipidemia, smoking and CAD severity. A *P* value of < 0.05 (2-tailed) was considered statistically significant. All statistical analyses were conducted using SPSS version 18.0 (IBM Co., Armonk, NY, USA).

## Results

**Figure 1:**
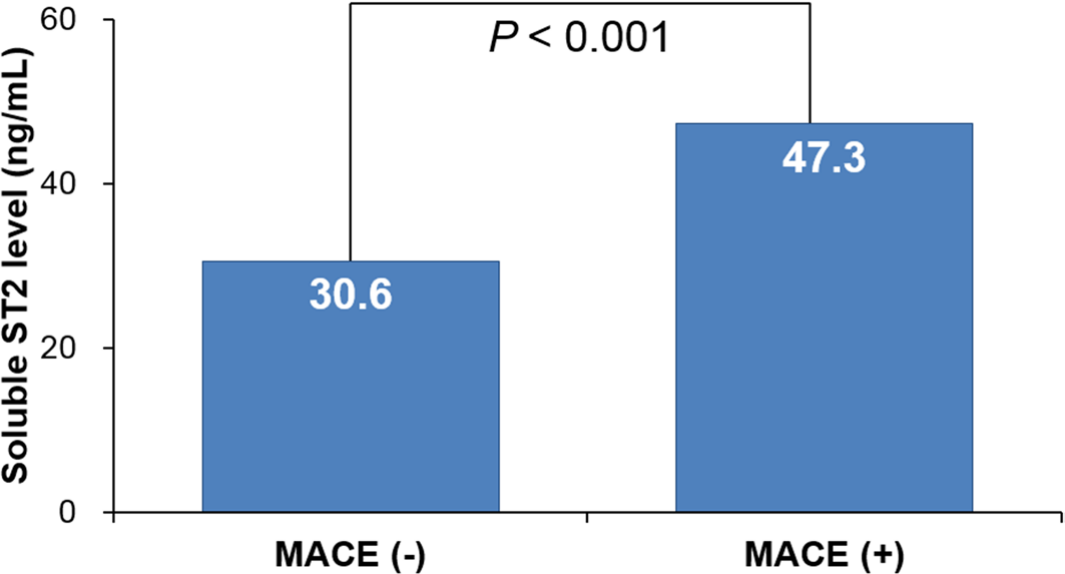
Soluble ST2 levels in patients with and without events. *MACE* major adverse cardiac event.

**Figure 2:**
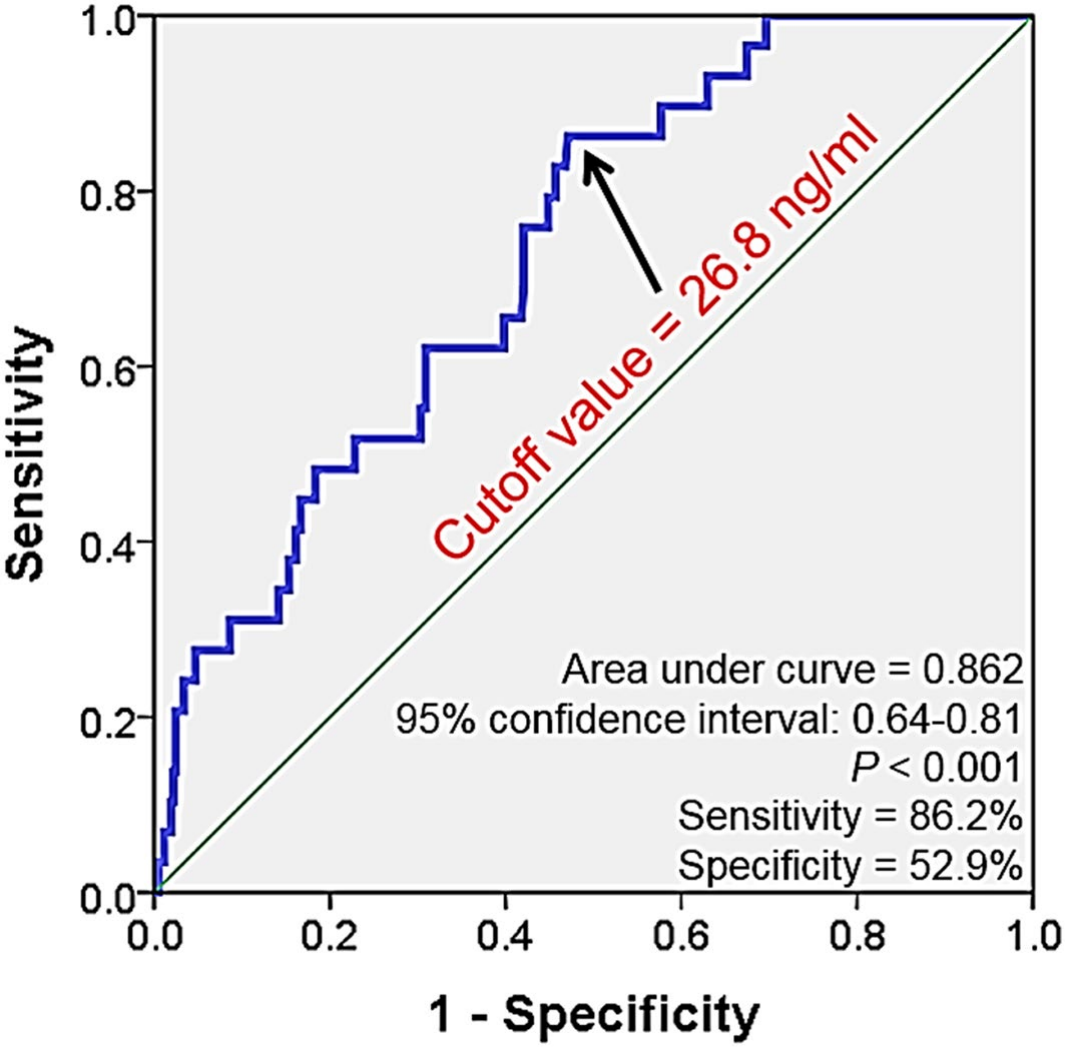
Receiver operating curve analysis showing cutoff value of soluble ST predicting major adverse cardiac event.

**Figure 3:**
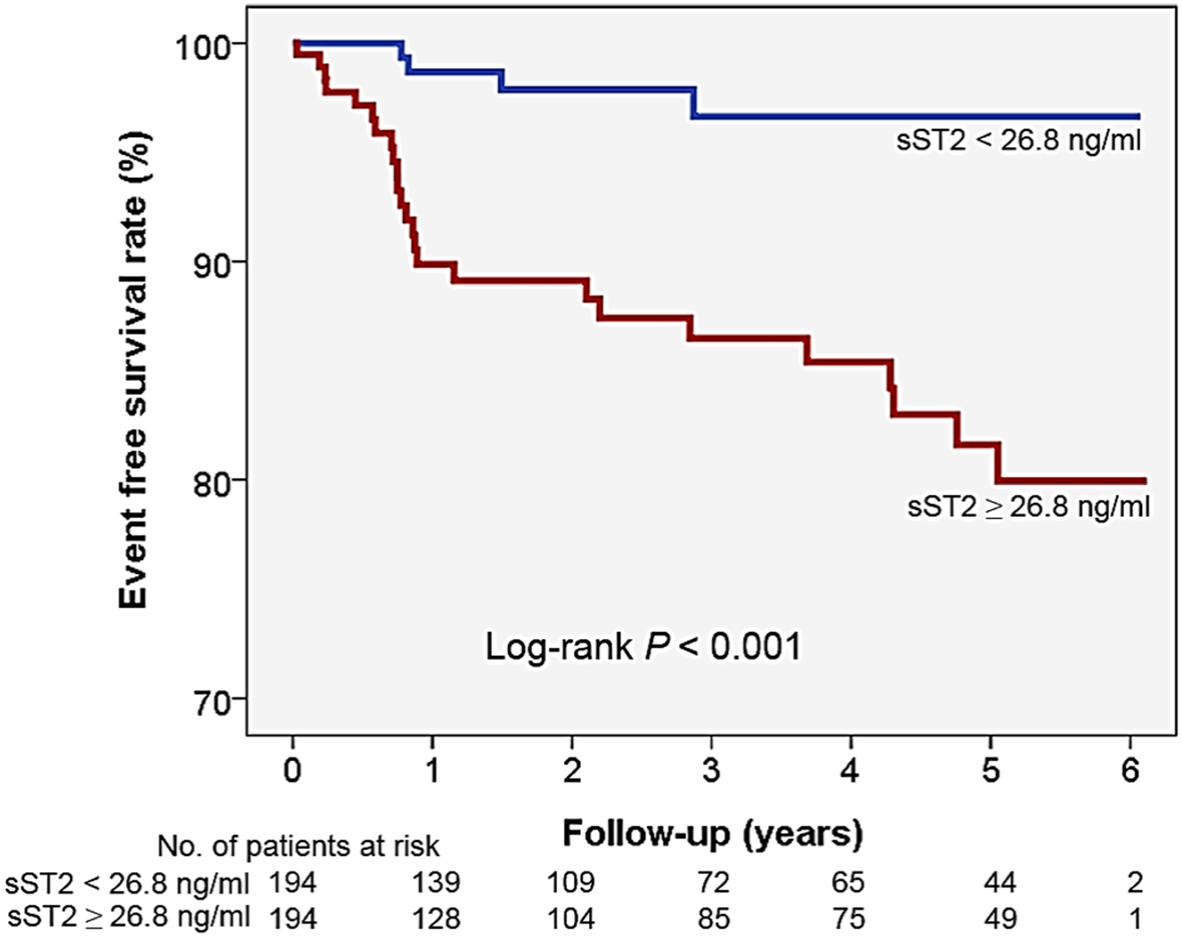
Event free survival rate according to soluble ST2 value.

During the mean follow-up period of 1013 ± 760 days (median, 884 days; interquartile range, 285–1804 days), 29 cases of MACE (7.5%) were documented. There were 4 cases of cardiac death, 3 cases of non-fatal AMI, 23 cases of coronary revascularization, and 4 cases of ischemic stroke. The baseline characteristics of study patients with and without MACE are shown in Table 1. There were no significant differences in most of the clinical characteristics including age, sex, blood pressure, risk factors and laboratory findings, as well as medications between patients with and without MACE. Only BMI and ICA findings were significantly different between the 2 groups: BMI was lower, and CAD was more severe in patients with MACE than those without MACE. Serum sST2 level was significantly higher in patients with MACE than those without (47.3 ± 32.3 vs. 30.6 ± 20.7 ng/ml, *P* < 0.001) (Fig. 1). The cut-off sST2 value of as 26.8 ng/ml predicting MACE was obtained in ROC curve analysis (area under curve, 0.862; 95% confidence interval, 0.64–0.81; sensitivity, 86.2%; specificity, 52.9%; *P* < 0.001) (Fig. 2). The MACE rates were significantly higher in patients with higher sST2 (≥ 26.8 ng/ml) than those with lower sST2 (< 26.8 ng/ml) (2.1% vs. 12.9%, *P* = 0.029), which is also demonstrated in Kaplan–Meier survival curves (log-rank *P* < 0.001) (Fig. 3). In multiple Cox regression model, sST2 was an independent predictor of MACE even after controlling for potential confounders (hazard ratio, 13.7; 95% confidence interval 1.80–104.60; *P* = 0.011) (Table 2). Patients were stratified into 2 groups according to the cutoff value of sST2 (= 26.8 ng/ml), and compared clinical characteristics between the 2 groups. Both groups did not relevantly differ in general clinical and laboratory parameters. However, patients with a higher sST2 level (≥ 26.8 ng/ml) group showed lower BMI, higher incidence of hypertension and lower LVEF compared to those with a lower sST2 level (< 26.8 ng/ml) (*P* < 0.05 for each) (Table 3).

**Table 1:** Baseline clinical characteristics of study patients.

Characteristic	With events (n = 29)	Without events (n = 359)	*P*
Age, years	67.8 ± 10.9	64.9 ± 10.6	0.162
Male sex, n (%)	21 (72.4)	226 (63.0)	0.308
Body mass index, kg/m^2^	23.4 ± 2.9	25.4 ± 3.6	0.004
Systolic blood pressure, mmHg	142 ± 26	134 ± 25	0.125
Diastolic blood pressure, mmHg	79.7 ± 14.6	79.0 ± 13.7	0.768
Heart rate, per minute	71.9 ± 12.2	71.5 ± 14.3	0.879
**Coronary risk factors, n (%)**			
Hypertension	20 (69.0)	232 (65.2)	0.679
Diabetes mellitus	12 (41.4)	107 (30.0)	0.201
Dyslipidemia	14 (48.3)	138 (38.7)	0.308
Smoking	7 (24.1)	86 (24.0)	0.982
**Clinical diagnosis, n (%)**			
Stable angina	13 (44.8)	209 (58.2)	0.161
Unstable angina	16 (55.2)	150 (41.8)	
**Major laboratory findings**			
Total-cholesterol, mg/dl	150 ± 31	148 ± 36	0.722
LDL cholesterol, mg/dl	85.9 ± 29.2	84.8 ± 32.7	0.867
HDL cholesterol, mg/dl	43.5 ± 12.9	41.3 ± 12.2	0.382
Triglyceride, mg/dl	108 ± 53	118 ± 78	0.517
Estimated GFR, ml/min/1.73 m^2^	83.5 ± 33.0	78.0 ± 23.4	0.241
LVEF, %	62.1 ± 7.6	64.0 ± 9.6	0.314
**Results of ICA, n (%)**			
Insignificant	1 (3.4)	69 (20.1)	0.020
One vessel disease	8 (27.6)	102 (29.7)	
Two vessel disease	6 (20.7)	88 (25.6)	
Three vessel disease	14 (48.3)	85 (24.7)	
**Concomitant medications, n (%)**			
Antiplatelet	20 (68.9)	232 (64.6)	0.704
Beta-blocker	15 (51.7)	177 (49.3)	0.802
RAS blocker	16 (55.2)	170 (47.4)	0.418
Statin	22 (75.9)	253 (70.9)	0.568

*LDL* low-density lipoprotein, *HDL* high-density lipoprotein, *GFR* glomerular filtration rate, *LVEF* left ventricular ejection fraction, *ICA* invasive coronary angiography, *RAS* renin-angiotensin system.

**Table 2:** Independent association between soluble ST2 and clinical outcomes.

Variable	Hazard ratio	95% confidence interval	*P*
Age ≥ 65 years	0.99	0.34–2.88	0.991
Female sex	0.75	0.24–2.30	0.616
Body mass index ≥ 25 kg/m^2^	0.33	0.11–0.98	0.047
Hypertension	0.58	0.18–1.90	0.370
Diabetes mellitus	2.30	0.77–6.86	0.133
Dyslipidemia	2.60	0.95–7.10	0.062
Smoking	0.99	0.31–3.10	0.994
Multi-vessel disease	3.15	0.88–11.27	0.078
Soluble ST2 ≥ 26.8 ng/ml	13.7	1.80–104.60	0.011

**Table 3:** Clinical characteristics of study patients according to soluble ST2 level.

Characteristic	sST2 ≥ 26.8 ng/ml (n = 194)	sST2 < 26.8 ng/ml (n = 194)	*P*
Age, years	66.0 ± 10.5	64.3 ± 10.7	0.126
Body mass index, kg/m^2^	24.9 ± 3.9	25.6 ± 3.1	0.049
Systolic blood pressure, mmHg	135 ± 26	134 ± 23	0.590
Diastolic blood pressure, mmHg	80.1 ± 14.1	77.9 ± 13.3	0.127
Heart rate, per minute	72.0 ± 15.2	71.1 ± 13.0	0.515
**Coronary risk factors, n (%)**			
Hypertension	137 (71.0)	115 (59.9)	0.022
Diabetes mellitus	61 (31.6)	58 (30.1)	0.741
Dyslipidemia	69 (35.8)	83 (43.0)	0.145
Smoking	49 (25.3)	44 (22.7)	0.552
**Clinical diagnosis, n (%)**			
Stable angina	94 (48.5)	82 (42.2)	0.324
Unstable angina	100 (51.5)	112 (57.8)	
**Major laboratory findings**			
Total-cholesterol, mg/dl	148 ± 37	147 ± 34	0.829
LDL cholesterol, mg/dl	85.6 ± 31.8	84.2 ± 33.0	0.692
HDL cholesterol, mg/dl	41.9 ± 14.0	41.1 ± 10.2	0.554
Triglyceride, mg/dl	110 ± 73	125 ± 78	0.081
Estimated GFR, ml/min/1.73m^2^	77.8 ± 26.8	78.9 ± 21.5	0.678
LVEF, %	62.4 ± 11.1	65.2 ± 7.3	0.006
**Results of ICA, n (%)**			
Insignificant	31 (16.6)	39 (21.0)	0.393
One-vessel disease	51 (27.3)	59 (31.7)	
Two-vessel disease	51 (27.3)	43 (23.1)	
Three-vessel disease	54 (28.9)	45 (24.2)	
**Concomitant medications, n (%)**			
Antiplatelet	128 (65.9)	124 (63.9)	0.815
Beta-blocker	97 (50.0)	95 (49.0)	0.839
RAS blocker	96 (49.5)	90 (46.4)	0.542
Statin	141 (73.1)	134 (69.4)	0.431

*LDL* low-density lipoprotein, *HDL* high-density lipoprotein, *GFR* glomerular filtration rate, *LVEF* left ventricular ejection fraction, *ICA* invasive coronary angiography, *RAS* renin-angiotensin system.

Sex-specific analysis was performed and main results were as follows: (1) serum sST2 level was higher in men than in women (33.4 ± 25.9 vs. 29.2 ± 13.1 ng/ml, *P* = 0.037), (2) Kaplan–Meier survival analyses showed that higher baseline sST2 levels were associated with worse clinical outcomes in both men and women (log-rank *P* < 0.05 for each sex) (Supplementary Figure S1), and (3) in multivariable analyses, serum sST2 level was associated clinical outcome only in men but not in women (Supplementary Table S1).

## Discussion

In the present study, a median follow-up period of 884 days of MACE in stable CAD patients revealed that: (1) the sST2 level was significantly higher in patients with MACE than those without, (2) MACE free survival rates were significantly lower in patients with higher sST2 (≥ 26.8 ng/ml) than those with lower sST2 (< 26.8 ng/ml), and (3) sST2 at baseline was independently associated with MACE even after controlling for other cardiovascular risk factors. These findings may extend the role of sST2 from heart failure and AMI to stable CAD, thereby widening the spectrum of clinical usefulness of sST2 as a prognostic marker.

### Prognostic value of sST2 in stable CAD

Although there have been many studies showing that sST2 is a useful biomarker for the prediction of cardiovascular events in patients with heart failure^5–7^ and AMI^8–10^, reported data on the prognostic value of sST2 in stable CAD patients are still scarce. Recently, evidence began to accumulate that sST2 also has value in stable CAD. The prognostic value of sST2 in low-risk patents with stable CAD has been evaluated in 2 other recent studies. Dieplinger et al.^12^ showed that baseline sST2 is an independent predictor of long-term all-cause and cardiovascular mortality in 1345 patients with stable CAD. Demyanets et al.^13^ demonstrated, in a study of 178 patients with stable angina, that sST2 significantly predicts death, myocardial infarction and re-admission for cardiac causes in a mean of 43-month follow-up duration. These findings are in line with ours, and extend the prognostic value of sST2 to patients with stable CAD. Moreover, we suggested a sST2 value of 28.7 ng/ml as the cut-off value for predicting MACE in stable CAD patients, which is consistent with that of a previous study^12^. This practical information would be valuable for the clinicians in the management of patients with stable CAD.

The mechanism involved in sST2 pathophysiology in stable CAD remains unclear, but a possible hypothesis can be suggested. As shown in our study, many risk factors were related to higher sST2 levels including lower BMI, and higher incidence of hypertension, and lower LVEF. Therefore, it is plausible that prognostic value of sST2 was reflected by integration of these risk factors.

### Clinical implications

Recently, potential circulating biomarkers that aid in risk assessment of patients with cardiovascular disease have received considerable attention. Indeed, several biomarkers such as natriuretic peptide^14^ and C-reactive protein^15^ were identified as having additional predictive power, and they are applied to clinical practice. A novel biomarker, sST2, also showed powerful prognostic value in heart failure^5–7^ and myocardial infarction^8–10^. However, the role of sST2 has been poorly characterized in patients with stable CAD. Although stable CAD is a more common clinical presentation among patients undergoing PCI^16^, it has been reported that a substantial proportion of patients with stable CAD receive inadequate treatment^17^. Traditional risk factors such as age, sex, hypertension, diabetes mellitus, dyslipidemia and smoking have remained the cornerstone of risk stratification^18^. However, it has been suggested that prognostic power of these risk factors for future cardiovascular events was weaker than that of biomarkers^19,20^. Therefore, new biomarkers for optimal risk stratification in patients with stable CAD should be emphasized. Our study data, taken together with recent data on sST2^12,13^, support the concept that sST2 could be applied to stable CAD as a useful biomarker. Measurement of this biomarker may be good help in identifying high-risk patients who are at risk of cardiovascular events and will have benefit from intensified management. Moreover, combining sST2 value with traditional risk factors may improve risk prediction ability. Despite the clinical association confirmed in this study, further studies are needed to investigate how sST2 can be used to guide therapy in patients with stable CAD.

### Obesity and better clinical outcome

In our multivariable analysis, obesity was associated with better clinical outcome. Despite evidence of a positive association between obesity and increased cardiovascular morbidity, some studies have shown a better clinical outcome in overweight and obese patients. This “obesity paradox” can be observed in a variety of clinical settings including stable CAD. There are reports describing a protective effect of obesity in stable patients undergoing PCI^21–23^, which is in line with our study finding.

### Study limitations

Several limitations of this study have to be addressed. First, small number of study patients and the low event rate might be associated with reduced statistical power. Despite this limitation, sST2 provided stronger results than known other risk factors, highlighting the strong prognostic merit of this biomarker. Secondly, serial measurements of ST2 would be valuable in the assessment of ST2 changes in response to therapy^24^. However, only a single measurement of ST2 was performed in our study. Lastly, our results pertain only to patients undergoing PCI, and thus cannot be generalized to all stable CAD patients.

## Conclusion

Elevated levels of baseline sST2 may be associated with an increased risk of adverse cardiovascular events in stable CAD patients undergoing ICA. Further investigations with a larger sample size are needed to confirm the prognostic value of sST2 in a broader group of stable CAD patients.

## Supplementary Information

Supplementary Information.

## Data Availability

All data generated or analyzed during this study are included in this article.

## Abbreviations

AMI: Acute myocardial infarction
BMI: Body mass index
CAD: Coronary artery disease
eGFR: Estimated glomerular filtration rate
HDL: High-density lipoprotein
ICA: Invasive coronary angiography
IRB: Institutional Review Board
LDL: Low-density lipoprotein
MACE: Major adverse cardiac events
SD, PCI: Percutaneous coronary intervention
ROC: Receiver operating characteristic standard deviation
sST2: Soluble ST2

## References

[1] Lozano R (2010). Global and regional mortality from 235 causes of death for 20 age groups in 1990 and 2010: A systematic analysis for the global burden of disease study 2010. Lancet.

[2] Leal J, Luengo-Fernández R, Gray A, Petersen S, Rayner M (2006). Economic burden of cardiovascular diseases in the enlarged European Union. Eur. Heart J..

[3] Morrow DA (2007). National academy of clinical biochemistry laboratory medicine practice guidelines: Clinical characteristics and utilization of biochemical markers in acute coronary syndromes. Circulation.

[4] Weinberg EO (2002). Expression and regulation of ST2, an interleukin-1 receptor family member, in cardiomyocytes and myocardial infarction. Circulation.

[5] Manzano-Fernández S, Mueller T, Pascual-Figal D, Truong QA, Januzzi JL (2011). Usefulness of soluble concentrations of interleukin family member ST2 as predictor of mortality in patients with acutely decompensated heart failure relative to left ventricular ejection fraction. Am. J. Cardiol..

[6] Weinberg EO, Shimpo M, Hurwitz S, Tominaga S, Rouleau JL, Lee RT (2003). Identification of serum soluble ST2 receptor as a novel heart failure biomarker. Circulation.

[7] Ky B (2011). High-sensitivity ST2 for prediction of adverse outcomes in chronic heart failure. Circ. Heart Fail..

[8] Shimpo M (2004). Serum levels of the interleukin-1 receptor family member ST2 predict mortality and clinical outcome in acute myocardial infarction. Circulation.

[9] Dhillon OS, Narayan HK, Quinn PA, Squire IB, Davies JE, Ng LL (2011). Interleukin 33 and ST2 in non-ST-elevation myocardial infarction: Comparison with global registry of acute coronary events risk scoring and NT-proBNP. Am. Heart J..

[10] O'Malley RG (2014). Prognostic performance of multiple biomarkers in patients with non-ST-segment elevation acute coronary syndrome: Analysis from the MERLIN-TIMI 36 trial (Metabolic Efficiency With Ranolazine for Less Ischemia in Non-ST-Elevation Acute Coronary Syndromes-Thrombolysis In Myocardial Infarction 36). J. Am. Coll. Cardiol..

[11] Levine GN (2012). 2011 ACCF/AHA/SCAI Guideline for Percutaneous Coronary Intervention: executive summary: a report of the American College of Cardiology Foundation/American Heart Association Task Force on Practice Guidelines and the Society for Cardiovascular Angiography and Interventions. Catheter. Cardiovasc. Interv..

[12] Dieplinger B (2014). Increased soluble ST2 predicts long-term mortality in patients with stable coronary artery disease: Results from the Ludwigshafen risk and cardiovascular health study. Clin. Chem..

[13] Demyanets S (2014). Soluble ST2 and interleukin-33 levels in coronary artery disease: Relation to disease activity and adverse outcome. PLoS ONE.

[14] Tsutamoto T (1997). Attenuation of compensation of endogenous cardiac natriuretic peptide system in chronic heart failure: Prognostic role of plasma brain natriuretic peptide concentration in patients with chronic symptomatic left ventricular dysfunction. Circulation.

[15] Danesh J (2004). C-reactive protein and other circulating markers of inflammation in the prediction of coronary heart disease. N. Engl. J. Med..

[16] Agema WR (2004). Current PTCA practice and clinical outcomes in the Netherlands: The real world in the pre-drug-eluting stent era. Eur. Heart J..

[17] Wiest FC, Bryson CL, Burman M, McDonell MB, Henikoff JG, Fihn SD (2004). Suboptimal pharmacotherapeutic management of chronic stable angina in the primary care setting. Am. J. Med..

[18] Goff DC (2014). 2013 ACC/AHA guideline on the assessment of cardiovascular risk: A report of the American College of Cardiology/American Heart Association task force on practice guidelines. Circulation.

[19] Beatty AL (2015). Traditional risk factors versus biomarkers for prediction of secondary events in patients with stable coronary heart disease: From the Heart and Soul study. J. Am. Heart. Assoc..

[20] Kleber ME (2014). Evolving biomarkers improve prediction of long-term mortality in patients with stable coronary artery disease: The BIO-VILCAD score. J. Int. Med..

[21] Gruberg L (2002). The impact of obesity on the short-term and long-term outcomes after percutaneous coronary intervention: The obesity paradox?. J. Am. Coll. Cardiol..

[22] Hastie CE (2010). Obesity paradox in a cohort of 4880 consecutive patients undergoing percutaneous coronary intervention. Eur. Heart J..

[23] Romero-Corral A (2006). Association of bodyweight with total mortality and with cardiovascular events in coronary artery disease: A systematic review of cohort studies. Lancet.

[24] Weir RA (2010). Serum soluble ST2: A potential novel mediator in left ventricular and infarct remodeling after acute myocardial infarction. J. Am. Coll. Cardiol..

