# Supplementary material for: Prognostic value of serum soluble ST2 in stable coronary artery disease: a prospective observational study
Source: Sci Rep. 2021 Jul 26;11:15203. doi: 10.1038/s41598-021-94714-3 (PMC8313553; doi:10.1038/s41598-021-94714-3)
Supplement: Supplementary file 1 — Supplementary Information. [file 41598_2021_94714_MOESM1_ESM.docx]

**Supplementary material**

**Supplementary Figure S1. Kaplan-Meier survival curve analyses showing event free survival rates according to baseline serum ST2 level in men and women**


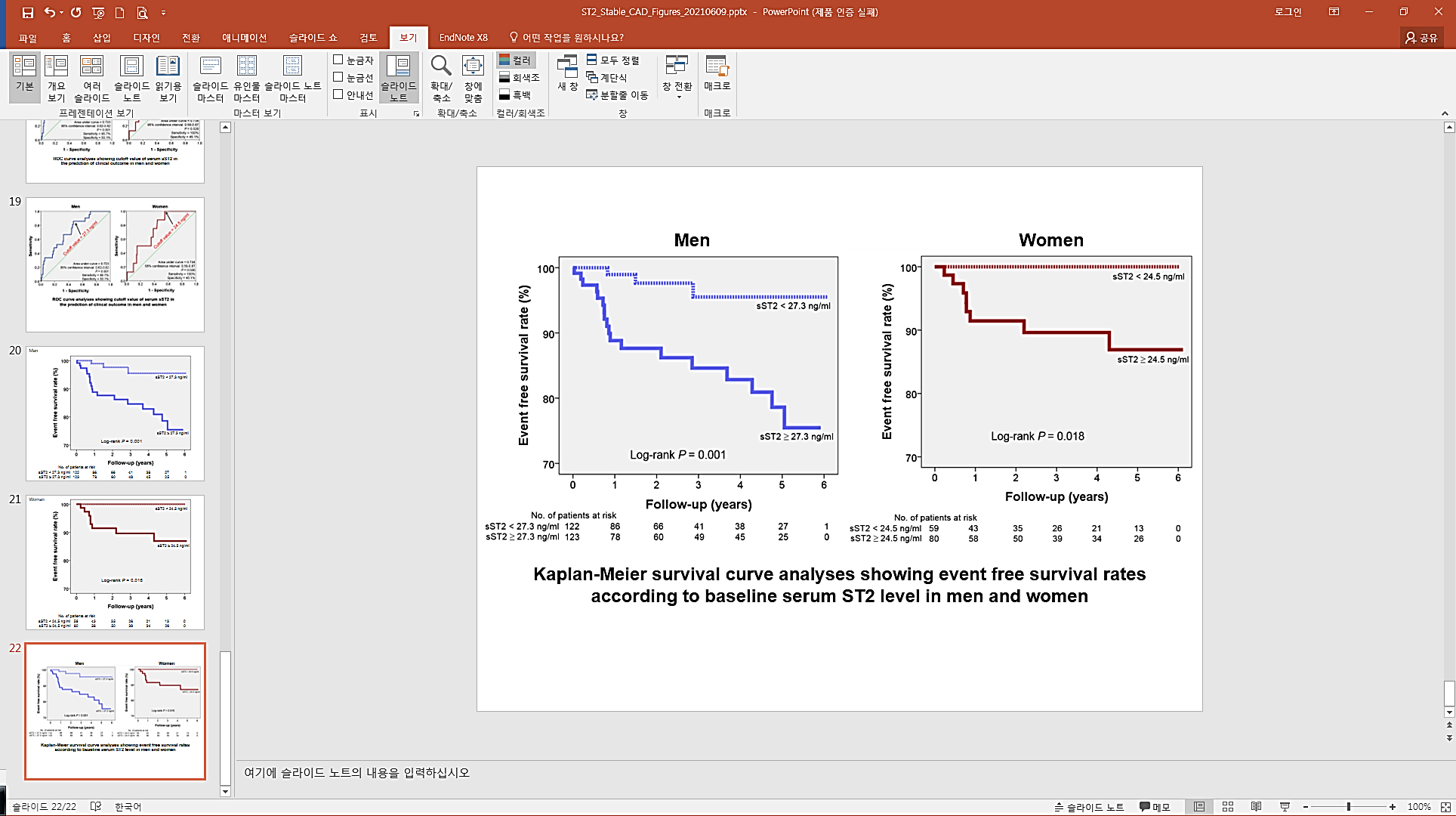


**Supplementary Table S1. Independent association between soluble ST2 and clinical outcomes in men and women**

| **Variable** | **Hazard ratio** | **95% confidence interval** | ***P*** |
| --- | --- | --- | --- |
| *Men* |  |  |  |
| Age ≥ 65 years | 1.23 | 0.39-3.84 | 0.720 |
| Body mass index ≥ 25 kg/m^2^ | 0.21 | 0.05-0.79 | 0.022 |
| Hypertension | 0.54 | 0.15-1.86 | 0.334 |
| Diabetes mellitus | 1.61 | 0.48-5.32 | 0.432 |
| Multi-vessel disease | 2.00 | 0.62-14.45 | 0.169 |
| Soluble ST2 ≥ 27.3 ng/ml | 11.40 | 1.47-88.16 | 0.002 |
| *Women* |  |  |  |
| Age ≥ 65 years | 0.20 | 0.11-3.90 | 0.294 |
| Body mass index ≥ 25 kg/m^2^ | 0.99 | 0.15-6.58 | 0.997 |
| Hypertension | 0.69 | 0.19-2.34 | 0.540 |
| Diabetes mellitus | 6.06 | 0.61-60.21 | 0.124 |
| Multi-vessel disease | 2.90 | 0.26-33.50 | 0.380 |
| Soluble ST2 ≥ 24.5 ng/ml | 47.92 | 0.14-16117.19 | 0.192 |
